# Total Number of Lymph Nodes in Oncologic Resections, Is There More to Be Found?

**DOI:** 10.1007/s11605-015-2764-9

**Published:** 2015-02-18

**Authors:** Kirsten J. de Burlet, Mari F. C. M. van den Hout, Hein Putter, Vincent T. H. B. M. Smit, Henk H. Hartgrink

**Affiliations:** 1 Department of Surgery, K6-50, Leiden University Medical Center, Albinusdreef 2, 2333 ZA Leiden, The Netherlands; 2 Department of Pathology, Leiden University Medical Center, Leiden, The Netherlands; 3 Department of Medical Statistics, Leiden University Medical Center, Leiden, The Netherlands

**Keywords:** Lymph node count, Staging, Oncologic resections, TNM classification

## Abstract

Pathologic staging of oncologic specimens includes the identification of the accurate lymph node status. Retrieving more lymph nodes leads to a more reliable N0 status in the TNM classification. The aim of this prospective study was to evaluate whether more lymph nodes can be retrieved from oncologic resection specimens when more time is invested in the search and if this contributes to a more reliable N-status in the individual patient. A total of 67 gastrointestinal oncologic specimens were reexamined for additional lymph nodes. The mean number of lymph nodes collected in the prospective group was compared against two retrospective groups, one before minima for lymph node counts were set (retrospective group 1) and one after (retrospective group 2). More lymph nodes were dissected per specimen in the prospective group (24.1 lymph nodes), compared to the retrospective group (14.3 lymph nodes, *P* = <0.001). During the study period, more patients were diagnosed as pN+ compared to the two retrospective groups (62.7 vs. 47.8 % respectively, *P* = 0.082). Significantly more lymph nodes can be found in oncologic specimens when more time is invested in the search. This will result in more accurate staging of the tumor.

## Introduction

Accurate pathologic staging of oncologic specimens includes the identification of lymph node metastases. Retrieving more lymph nodes leads to a more reliable N status, especially the pN0 status in the TNM classification, thereby improving the accuracy of 5-year prognosis.^1^^–^ 3 Moreover, the finding of even a single lymph node metastasis has, in some tumor types, major implications for adjuvant therapy.

Concerning the number of lymph nodes that should be evaluated, oncologic guidelines like the American Joint Committee on Cancer (AJCC, www.cancerstaging.org) suggest more than 12 lymph nodes dissected per colorectal specimen and more than 15 lymph nodes for gastric cancer.^2^^–^ 6 There are no guidelines with cutoff values for the extent of lymphadenectomy regarding esophagectomy or pancreaduodenectomy (Whipple) specimens.

Several studies have shown that if more lymph nodes are found in a pT1–3N0M0 cancer patient, it will lead to a longer 5-year survival rate. This is reported for several types of cancer, like esophageal, stomach, colon, and rectal cancer.^3^^–^ 7 The most likely explanation for the observed association between increased number of negative lymph nodes and improved survival is incorrect staging of patients with missed positive lymph nodes due to the low total number of investigated lymph nodes. So, the reliability of the pN0 status will increase when more node negative lymph nodes are found.^5^^–^ 11

An extended lymphadenectomy not only includes the enlarged lymph nodes. It is well known that severely enlarged lymph nodes, especially the nodes that have lost their ellipsoid contour, often contain tumor. However, lymph node metastases are not only found in large lymph nodes. In fact, several studies have shown that more than half of the retrieved lymph nodes containing tumor deposits are smaller than 5 mm.^12^^–^ 15

Variables that may influence lymph node retrieval are potentially related to the patient, the surgeon, the pathologist, and neo-adjuvant regimens. Several studies have tried to investigate these variables in more detail. Literature suggests that the largest variation in the number of lymph nodes retrieved in surgical specimens is accounted for by differences between pathologists and not between surgeons.^16^^–^ 20 Still, there is an ongoing discussion between both, especially when small numbers of lymph nodes are encountered. The surgeon may feel that there are more lymph nodes to be found in the surgical specimen, while the pathologists may state that the surgeon’s resection was not complete enough.

In an attempt to shed light on this discussion, both specialists agreed to participate in the study format as designed below.

## Materials and Methods

Between November 2011 and June 2012, all specimens from the following surgical oncologic resections were prospectively collected in the Leiden University Medical Center: esophagus, stomach (partial and total), pancreas (pancreaticoduodenectomy according to Whipple), colon, and rectum. Excluded were specimens which, after microscopic evaluation, were diagnosed as benign or gastrointestinal stromal tumors (GIST).

All patients underwent a potentially curative resection, and all of them were preoperatively discussed in a multidisciplinary team with radiologists, oncologists, radiotherapists, surgeons, and pathologists. In these weekly meetings, decisions were made about the type of resection and, if relevant, (neo-)adjuvant treatment. Current protocol in the Leiden University Medical Center (based on national guidelines) recommends neo-adjuvant chemoradiation therapy (CRT) for esophageal tumors, neo-adjuvant chemotherapy (CT) for gastric cancer, and neo-adjuvant radiotherapy (RT) for T2–T4 rectal carcinomas. Neo-adjuvant therapy was renounced if a patient was unfit for the recommended treatment. Surgical techniques and protocols for (neo-)adjuvant treatment did not change during the study period.

Every specimen was routinely evaluated by the pathologist. This included a complete lymph node search of all the resected tissue by the pathology resident after overnight formalin fixation and microscopic evaluation of the lymph nodes by the resident and senior pathologist. During the study period, the mean time invested for this basic lymph node search was 20 min per specimen. Consequently, during the same week, a researcher (KdB) searched for additional lymph nodes in the residual macroscopic tissue for exactly 20 min. The number of potential lymph nodes retrieved was recorded every 5 min by the researcher. These included additional lymph nodes that were routinely processed and evaluated by the resident and senior pathologist and reported in the final pathology report. During the study period, mainly two pathology residents examined the specimens. One resident was involved from November until the end of December 2011 and the other from January until June 2012. On occasion, another resident pathologist substituted.

According to the Dutch rules for research for this study, no approval from the ethics committee was deemed necessary as only routine diagnostics were extended. Therefore, no informed consent was obtained from patients. For the researcher, all samples were handled in a coded fashion according to National Ethical Guidelines.

In order to evaluate the number of lymph nodes found in oncologic specimens in time, we retrospectively searched operation files for operated patients and matched them by organ and neo-adjuvant therapy to the prospective group.

Ultimately, retrospective group 1 consisted of surgical specimens from which lymph node data were collected between January and December 2008. Retrospective group 2 consisted of surgical specimens retrospectively collected between September 2010 and November 2011. These periods were deliberately chosen before and after 2009 because in that year, minima were set for the number of lymph nodes dissected in colon and stomach resections to improve accurate staging (AJCC). In this manner, we could also evaluate the development of lymph node counts under different protocols.

The database was statistically analyzed in SPSS (version 20). Numerical variables are summarized as mean (SD) or range. The differences between the retrospective and prospective group characteristics (age and gender) were assessed using the independent samples *t* test for age and chi-square test for gender. Differences between groups with respect to number of lymph nodes found were assessed using the independent samples *t* test (comparisons between retrospective 1 and 2, between retrospective 2 and prospective A) and the paired samples *t* test (comparison between prospective groups A and B).

## Results

The prospectively examined group consisted of a total of 67 oncologic specimens from patients operated in the Leiden University Medical Center. These specimens were categorized by six different types of resections performed between November 2011 and June 2012 (Table 1). The mean age of the patients was 66.3 years, and 57 % of the patients were male. The mean age and gender did not differ significantly between the retrospective and prospective groups. As shown in Table 1, 25 patients received neo-adjuvant therapy. Twelve of them received neo-adjuvant chemoradiotherapy (CRT). Eleven were diagnosed with esophageal cancer and one with gastric cancer. The latter was preoperatively diagnosed as esophageal cancer, but after resection, the tumor proved to be of gastric origin. Eleven patients with a gastric tumor received neo-adjuvant chemotherapy (CT), and two patients with a rectal tumor received neo-adjuvant radiotherapy (RT) (5 × 5 Gy).

**Table 1:** Specimen characteristics

	Esophagus (*n* = 11)	Partial gastric (*n* = 10)	Total gastric (*n* = 8)	Pancreas (*n* = 25)	Colon (*n* = 9)	Rectum (*n* = 4)	Total (*n* = 67)
Neo-adjuvant treatment, *n* (%)							
CT	0 (0 %)	5 (50 %)	6 (75 %)	0 (0 %)	0 (0 %)	0 (0 %)	11 (16.4 %)
RT	0 (0 %)	0 (0 %)	0 (0 %)	0 (0 %)	0 (0 %)	2 (50 %)	2 (3.0 %)
CRT	11 (100 %)	0 (0 %)	1 (12.5 %)	0 (0 %)	0 (0 %)	0 (0 %)	12 (17.9 %)
Tumor, *n* ( %)							
T1	2 (18.2 %)	1 (10.0 %)	2 (25.0 %)	4 (15.4 %)	2 (22.2 %)	0 (0 %)	11 (16.4 %)
T2	6 (54.5 %)	1 (10.0 %)	3 (37.5 %)	5 (20.0 %)	0 (0 %)	2 (50.0 %)	17 (25.4 %)
T3	2 (18.2 %)	7 (70.0 %)	3 (37.5 %)	16 (64.0 %)	6 (66.7 %)	2 (50.0 %)	36 (53.7 %)
T4	1 (9.1 %)	1 (10.0 %)	0 (0 %)	0 (0 %)	1 (11.1 %)	0 (0 %)	3 (4.5 %)
Node, *n* (%)							
N0	3 (27.3 %)	2 (20.0 %)	4 (50.0 %)	9 (36.0 %)	4 (44.4 %)	3 (75.0 %)	25 (32.8 %)
N1	6 (54.5 %)	4 (40.0 %)	2 (25.0 %)	16 (64.0 %)	4 (44.4 %)	1 (25.0 %)	33 (49.3 %)
N2	2 (18.2 %)	1 (10.0 %)	2 (25.0 %)	0 (0 %)	1 (11.1 %)	0 (0 %)	6 (9.0 %)
N3	0 (0 %)	3 (30.0 %)	0 (0 %)	0 (0 %)	0 (0 %)	0 (0 %)	3 (4.5 %)
Total, *n* (%)	11	10	8	25	9	4	67

Neo-adjuvant treatment: CT = chemotherapy, RT = radiotherapy, and CRT = chemotherapy and radiotherapy. Tumor and node according to TNM classification of the different specimens. Resection: R0 complete resection, R1 microscopic incomplete resection and R2 macroscopic incomplete resection

Tumor and node classification in this specimen cohort is shown in Table 1. Most of the resected specimens were pT2 or pT3 tumors (79.3 %). Twenty-five patients were classified as a pN0 tumor.

The mean number, standard deviation (SD), and range of lymph nodes dissected for each type of specimen are shown in Table 2. Lymph node count was significantly different between all groups. In retrospective group 1, before the protocol with minimal number of lymph nodes to be retrieved was implemented, *fewer* lymph nodes were collected (*P* = <0.001). Finding none or one lymph node in an oncologic specimen was reported more than once in this cohort. After minima were set (retrospective group 2), significantly more lymph nodes were evaluated (*P* = 0.001) and no pathology report includes less than five lymph nodes evaluated.

**Table 2:** Number of lymph nodes found per specimen and group

	Retrospective 1	Retrospective 2	Prospective
Esophagus	11.3 [4.2]	14.3 [5.0]	24.1 [7.4]*
	4–20	8–23	12–35
Partial gastric	10.3 [6.0]	18.4 [7.0]*	31.8 [9.2]*
	0–18	8–28	18–42
Total gastric	17.6 [7.3]	14.9 [5.3]	27.1 [9.3]*
	6–27	8–22	16–44
Pancreas	9.1 [5.5]	11.6 [3.7]	21.1 [7.1]*
	0–20	6–21	9–36
Colon	12.9 [4.5]	17.3 [5.2]*	24.1 [5.9]*
	5–19	11–28	14–31
Rectum	6.0 [5.0]	12.8 [5.9]	18.0 [6.4]
	1–11	5–19	14–31
Total	10.9 [7.0]	14.3 [5.5]*	24.1 [8.3]*
	0–27	5–28	9–44

Mean amount of lymph nodes found per group, [SD] and range

*P = <0.05, significant differences in mean lymph node count between the groups and per specimen (an independent sample *t* test was used)

Table 3 shows the mean total lymph node count, the malignant lymph node count, and the percentage of malignant lymph nodes. No significant differences were found in lymph node counts between the 25 patients treated with neo-adjuvant therapy and the 42 patients without neo-adjuvant therapy. The mean lymph node count in the two patients who received neo-adjuvant RT is lower compared to the other patients. These patients had a rectal tumor, and mean lymph nodes dissected for rectal specimens were overall lower in this cohort (see Table 1).

**Table 3:** Mean lymph node count dependent of tumor size and neo-adjuvant treatment

	Total lymph node count (mean)	Malignant lymph node count (mean)	Percentage malignant lymph nodes (%)
Neo-adjuvant treatment			
Nil (*n* = 42)	23.0	4.5	19.6
CT (*n* = 10)	30.8	3.6	11.7
RT (*n* = 2)	19.5	1.0	5.1
CRT (*n* = 13)	23.3	2.8	12.0
Response			
No neo-adjuvant treatment (*n* = 42)	23.0	4.3	18.7
No response (*n* = 3)	25.7	1.7	6.6
Partial (*n* = 19)	26.1	3.6	13.8
Good/complete (*n* = 3)	25.7	0	
Tumor			
T1 (*n* = 11)	23.4	1.2	5.1
T2 (*n* = 17)	24.3	2.8	11.5
T3 (*n* = 36)	23.7	4.7	19.8
T4 (*n* = 3)	30.0	11.0	36.7
Node			
N0 (*n* = 25)	24.1	0	0
N1 (*n* = 33)	23.6	4.0	16.9
N2 (*n* = 6)	23.8	8.0	33.6
N3 (*n* = 3)	29.7	25.0	84.2
Total (*n* = 67)	24.1	3.8	15.8

Neo-adjuvant treatment: CT = chemotherapy, RT = radiotherapy, and CRT = chemotherapy and radiotherapy. Tumor and node according to TNM classification of the different specimens. Resection: R0 complete resection, R1 microscopic incomplete resection, and R2 macroscopic incomplete resection

The mean number of lymph nodes retrieved did not differ between the levels of response to the given neo-adjuvant therapy. Patients with a complete or a good response in this cohort had no tumor-positive lymph nodes in the resected specimen.

pT4 tumors had a significant higher lymph node count compared to the pT1–3 tumors. The percentage of malignant lymph nodes in the specimens shows a gradual increase of the amount of tumor-positive nodes when the tumor grows in size.

Table 4 shows the number of patients diagnosed as N+ in the retrospective groups and the prospective group. More patients were diagnosed as N+ in the prospective group (32 vs. 42, *P* = 0.082). No difference was found between the two retrospective groups. There is no clear correlation between lymph node positivity (N+) and tumor origin.

**Table 4:** Differences in N status of the TNM classification in the study groups

	Retrospective 1	Retrospective 2	Prospective
N0	35 (52.2 %)	35 (52.2 %)	25 (37.3 %)
N+	32 (47.8 %)	32 (47.8 %)	42 (62.7 %)
Total	67 (100 %)	67 (100 %)	67 (100 %)

In Table 3, the amounts and percentages of total patients diagnosed with pN0 or pN+ per group are shown. More patients were diagnosed as N+ in the prospective group, and this was not significant (*P* = 0.082)

To evaluate the amount of dissected lymph nodes within the 20-min additive search, the cumulative mean number of probable lymph nodes found per 5 min was calculated. Adding 5- or 10-min search time increased the number of retrieved lymph nodes by 12 and 20 %, respectively. Lymph node retrieval decreased logarithmically in time. After 20 min, the cumulative mean number of extra lymph nodes retrieved was 6; this accounted for 25 % of the total number of dissected lymph nodes.

## Discussion

In this study, we found that when more time is invested in lymph node search, significantly more lymph nodes can be found in cancer resection specimens. Firstly, there is a clear improvement in lymph node retrieval after minima for the number of dissected lymph nodes per specimen were set in 2009. Secondly, we observed a significant difference between numbers of lymph nodes retrieved in the retrospective group 2 (after these minima were set) and the number of lymph nodes found in the prospective group. This leads to more patients diagnosed as N+ in the prospective cohort compared to the two retrospective cohorts; however, no significant difference was found (*P* = 0.082).

Other variables that may influence the number of lymph nodes found are related to the surgeon and possible neo-adjuvant therapy. If the surgical resection lacks sufficient lymphatic tissue, the pathologist will obtain fewer nodes. From studies on more extended lymph node dissections, we know that the surgical technique can make a great difference on the number of lymph nodes found. For gastric cancer, this was shown in a Dutch trial which showed an average of 17 lymph nodes found after a limited (D1) dissection and an average of 30 lymph nodes found after an extended (D2) dissection.^21^ In the current study, the same surgical resection techniques per specimen were used for all patients. Therefore, the surgical factor in the differences in lymph node retrieval found in this study is considered limited.

Some studies have found substantial variability between pathologists and the number of evaluated lymph nodes. Ostadi et al.^17^ evaluated factors which contributed to a difference in lymph node count in colorectal specimens. They concluded that most variation was accounted for by differences between pathologists. Evans et al.^18^ found no differences in lymph node harvest in colorectal cancer between individual surgeons but did find that there was a significant difference between reporting pathologists. Kuiper et al.^19^ reported that their laboratory technicians retrieved more lymph nodes than the pathologists, and Mekenkamp et al.^20^ found large differences in lymph node retrieval between various pathologists and between different laboratories. Based on the current study and the four studies cited above, it appears that the time invested in lymph node retrieval is the most crucial factor for adequate lymph node retrieval.^17^^–^ 20

From the pathologist’s point of view, there is a consensus that neo-adjuvant CRT may result in the retrieval of fewer lymph nodes, especially when conventional manual lymph node dissections are used.^20^^,^ 22 Mekenkamp et al.^20^ showed that after neo-adjuvant RT, fewer lymph nodes were retrieved in rectum specimens (6.9 vs. 8.5; *P* < 0.0001). In esophageal cancer, the same effect of CRT to lymph node retrieval has been shown (6.9 vs. 8.6; *P* = 0.026).^23^ RT is thought to decrease lymph node yield as a result of an immune response, rendering them undetectable because of RT-induced fibrosis. The shrinkage after RT makes lymph node retrieval more challenging. Nevertheless, in the above-mentioned studies, lymph node metastasis (and micro-metastasis) has been found in lymph nodes <0.3 cm. Thereby suggesting that, although lymph node evaluation is more challenging, it is crucial for accurate staging.^24^^–^ 26 In our study, there was no significant difference in lymph node counts between specimens treated with or without neo-adjuvant (C)RT. However, the groups were too small for accurate statistic analysis.

In order to facilitate large amounts of lymph node recovery, fat clearance techniques have been developed. Average lymph node counts over 50 have been reported when using these techniques. However, fat clearance techniques are not often used because they are time-consuming (1 to 5 weeks extra per specimen) and more expensive.^25^^,^ 26

Comparing the number of patients diagnosed as pN+ in our three groups shows an interesting result. The adjustment of guidelines did lead to an increased number of lymph nodes evaluated, but not to a change in percent of patients staged with pN+ disease. In the prospective group, however, we observed a clear stage migration since more patients were diagnosed as pN+ compared to the retrospective groups (62.7 vs. 47.8 %, respectively, *P* = 0.082). The more extensive search in the prospective groups may have led to this stage migration phenomenon.

The estimated average search time for lymph node retrieval by the pathology resident was approximately 20 min. The time for reexamination for additional lymph nodes was set at 20 min as a longer search seemed unfeasible for routine pathological practice. In the additional search, the largest number of extra lymph nodes was found during the first 5 min, and this number decreased logarithmically in time. Adding 5- or 10-min search time increased the number of retrieved lymph nodes by 12 and 20 %, respectively. Although data are insufficient to make firm conclusions, considering the estimated search time by the pathology resident, the optimal total time for lymph node retrieval may be around 30 min, although this may differ between oncologic specimens and origin, depending on the amount of fatty tissue.

There are some limitations to our study. Firstly, the number of extra lymph nodes found by the researcher was registered every 5 min, but the initial lymph node search by the pathology resident was not recorded in detail. Secondly, no data was collected on the outcome and the possible change in adjuvant treatment and prognosis. For certain tumor types, like pancreas carcinomas, the “stage migration” due to the extra dissected lymph nodes would not lead to a change in postoperative management. However, it will make a difference in prognosis, and therefore, it will still be relevant for the patient. For other tumor types, like colon cancer, it may alter adjuvant treatment.

Finally, the data from the two retrospective groups was collected via the electronic database of the hospital. This has its obvious retrospective data collection hazards, and for our study, specifically, there was no indication of the total time invested in lymph node search.

## Conclusion

Significantly more lymph nodes can be found in oncologic specimens when more time for searching is invested. This will improve accurate staging of the patients and could have important implications for (adjuvant) therapy and prognosis. The optimal time for retrieval of lymph nodes may be around 30 min, and the surplus value of retrieving decreases in time.
